# Short-term step reduction reduces citrate synthase activity without altering skeletal muscle markers of oxidative metabolism or insulin-mediated signaling in young males

**DOI:** 10.1152/japplphysiol.00650.2021

**Published:** 2021-11-04

**Authors:** Sophie J. Edwards, Brandon J. Shad, Ryan N. Marshall, Paul T. Morgan, Gareth A. Wallis, Leigh Breen

**Affiliations:** School of Sport, Exercise and Rehabilitation Sciences, University of Birmingham, Birmingham, United Kingdom; MRC-ARUK Centre for Musculoskeletal Ageing Research, University of Birmingham, Birmingham, United Kingdom

**Keywords:** insulin sensitivity, mitochondria, physical inactivity, skeletal muscle, step reduction

## Abstract

Mitochondria are critical to skeletal muscle contractile function and metabolic health. Short-term periods of step reduction (SR) are associated with alterations in muscle protein turnover and mass. However, the effects of SR on mitochondrial metabolism/muscle oxidative metabolism and insulin-mediated signaling are unclear. We tested the hypothesis that the total and/or phosphorylated protein content of key skeletal muscle markers of mitochondrial/oxidative metabolism, and insulin-mediated signaling would be altered over 7 days of SR in young healthy males. Eleven, healthy, recreationally active males (means ± SE, age: 22 ± 1 yr, BMI: 23.4 ± 0.7 kg·m^2^) underwent a 7-day period of SR. Immediately before and following SR, fasted-state muscle biopsy samples were acquired and analyzed for the assessment of total and phosphorylated protein content of key markers of mitochondrial/oxidative metabolism and insulin-mediated signaling. Daily step count was significantly reduced during the SR intervention (13,054 ± 833 to 1,192 ± 99 steps·day^−1^, *P* < 0.001). Following SR, there was a significant decline in maximal citrate synthase activity (fold change: 0.94 ± 0.08, *P* < 0.05) and a significant increase in the protein content of p-glycogen synthase (P-GS^S641^; fold change: 1.47 ± 0.14, *P* < 0.05). No significant differences were observed in the total or phosphorylated protein content of other key markers of insulin-mediated signaling, oxidative metabolism, mitochondrial function, or mitochondrial dynamics (all *P* > 0.05). These results suggest that short-term SR reduces the maximal activity of citrate synthase, a marker of mitochondrial content, without altering the total or phosphorylated protein content of key markers of skeletal muscle mitochondrial metabolism and insulin signaling in young healthy males.

**NEW & NOTEWORTHY** Short-term (7 day) step reduction reduces the activity of citrate synthase without altering the total or phosphorylated protein content of key markers of skeletal muscle mitochondrial metabolism and insulin signaling in young healthy males.

## Graphical abstract

**Figure:**
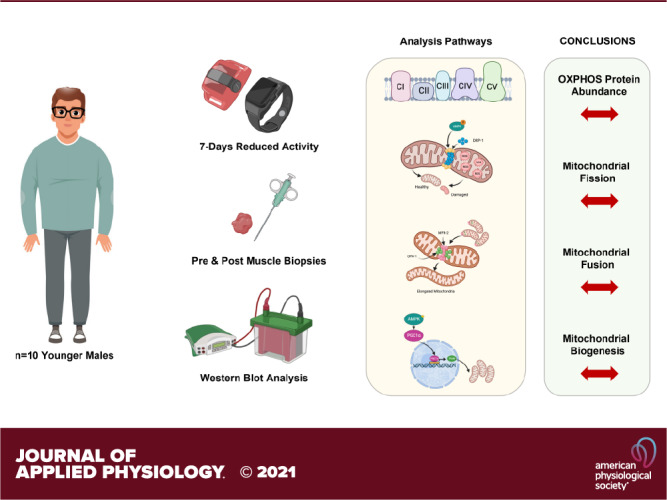

## INTRODUCTION

Musculoskeletal disuse occurs during illness (i.e., bed rest) and injury (i.e., limb immobilization). In addition to these periods of severe disuse, periods of reduced ambulation also occur throughout the human life span in times of illness and injury, as well as through gradual reductions in physical activity lifestyle habits with age in the absence of injury/illness. Periods of physical inactivity and reduced physical activity across the lifespan are accompanied by skeletal muscle atrophy (1–3), a decline in aerobic capacity (4), and a reduction in whole body insulin sensitivity (5–7). However, the mechanisms underpinning these responses remain to be fully elucidated and this is having a meaningful impact on the development of therapeutic interventions to improve patient treatment and outcome in attenuating skeletal muscle atrophy and impairments in muscle metabolism. Indeed, with the knowledge of specific proteins that may be altered during disuse, this may offer insights into specific targets for novel treatments during periods of disuse to offset muscle atrophy.

Disuse atrophy is underpinned by alterations to muscle protein turnover, primarily attributed to the reductions in myofibrillar protein synthesis rates (8–11). Recent evidence has also suggested that disuse atrophy is accompanied by alterations to mitochondrial metabolism and impaired aerobic capacity (12–16). Mitochondria are mechanically sensitive organelles (17) that are critical to contractile function (18), fuel utilization, and metabolic health (19, 20), which dictate aerobic capacity (21–23). Therefore, it is plausible that alterations in mitochondrial function during disuse may not only underpin reductions in aerobic capacity, but also contribute to muscle atrophy and impaired insulin sensitivity. In models of more severe musculoskeletal disuse (e.g., bed rest/immobilization), reductions in mitochondrial respiratory capacity (12), protein synthesis rates (11, 24), and oxidative phosphorylation (OXPHOS) complexes proteins (7, 12) have been noted in the first 14 days of disuse (25, 26). Furthermore, mitochondria morphology is ultimately dependent upon the fine balance between rates of mitochondrial fission and fusion. Preclinical models suggest that disuse events are accompanied by alterations to the mitochondrial dynamics (27), with the balance tilting toward mitochondrial fission (28), resulting in an increase of fragmented mitochondria (29–31). Although significant alterations in mitochondrial gene expression (e.g., *COX7A2*, *ATP5E*, and *MRPS36*) has been noted following 2 wk of step reduction (SR) in overweight and older adults (32), whether short-term SR in young adults results in similar alterations to muscle mitochondrial metabolism as more severe, longer-term models of disuse has not yet been explored.

Disuse-induced alterations in mitochondrial fragmentation and, thus, functioning have been implicated in the development of impaired insulin sensitivity, which is dampened during periods of severe disuse (5–7). Mitochondrial abnormalities are commonly observed in metabolically compromised patients (33) with an accompanying increase in reactive oxygen species (ROS) production, alterations in fuel utilization, and increases in mitochondrial fission often noted (34, 35). In preclinical models of severe disuse (i.e., hind limb unloading), mitochondrial dysfunction has been linked to alterations in fuel utilization through a shift toward glycolysis (36), which may underpin changes in whole body insulin sensitivity. Importantly, whole body insulin sensitivity appears to be preserved in insulin resistant models following a decline in ROS generation (34). Although muscle mitochondrial dysfunction may precede reductions in insulin sensitivity during periods of severe disuse (i.e., bed rest/immobilization), this has yet to be investigated in the context of SR. Therefore, we aimed to determine the impact of 7 days of SR on the expression of key skeletal muscle markers, of mitochondrial/oxidative metabolism, and insulin-mediated signaling in young healthy males. Our hypothesis was that 7-day SR in young males would *1*) reduce the maximal activity of oxidative enzymes (i.e., citrate synthase (CS) as a marker of mitochondrial content), *2*) reduce the total and phosphorylated protein content of key signaling intermediates involved in oxidative metabolism, *3*) promote mitochondrial fission (assessed via total and phosphorylated protein content of proteins involved in mitochondrial dynamics), and *4*) dampen the expression and phosphorylation of markers involved in the maintenance of skeletal muscle insulin sensitivity.

## METHODS

### Participants

The current study represents an extended retrospective analysis of a previously published study from our collective group (37). Eleven healthy, young males (means ± SE age: 22 ± 1 yr; BMI: 23.4 ± 0.7 kg·m^−2^) completed 7 days of SR. Before obtaining written informed consent, participants received oral and written information regarding the nature of the intervention and the possible risks of participation. All participants were deemed in good general health based on their responses to a general health questionnaire and were only excluded if they were diagnosed with existing health conditions (e.g., hypertension, diabetes), were a current smoker, and/or were suffering from musculoskeletal injury. If deemed eligible, participants were provided with an ActivePAL3 (PAL Technologies Ltd., Glasgow, UK) to assess step count for the 7 days before the step-reduction intervention. Participants that averaged <7,000 steps·day^−1^ were excluded from participation. Study approval was granted by the Research Ethics Service Committee West Midlands, Edgbaston, United Kingdom (Reference: 16/WM/0011), and the study was conducted in accordance to the Declaration of Helsinki. The intervention was registered at clinicaltrials.gov before data collection (Identifier: NCT02624011).

### Experimental Design

Participants were instructed to maintain their habitual physical activity levels for 7 days. Thereafter, participants were instructed to refrain from any structured physical activity and reduce their step count to ∼1,500 steps·day^−1^ for the 7-day period of SR. Activity was measured throughout the intervention using an ActivePAL3 accelerometer. During SR, participants were provided with visual feedback on daily step count through a hip worn pedometer (Yamax Digi-Walker SW-200). Following the 7-day period of habitual physical activity and again following the 7-day period of SR, participants reported to the laboratory at 0800 h in an overnight fasted state, where a muscle biopsy was obtained from the middle portion of the vastus lateralis using a suction-adapted percutaneous needle biopsy technique under local anesthesia (1% lidocaine). Muscle samples were freed from any visible nonmuscular material and rapidly frozen in liquid nitrogen before being stored at −80°C for future analysis. A more comprehensive description (including dietary control) of the experimental protocol can be found in our previous publication (37).

### Western Blotting

Snap-frozen muscle samples (∼50 mg) were manually homogenized on ice using a pestle in 10 μL of a standard extraction buffer per 1 mg tissue. Samples underwent centrifugation at 2,500 *g*, 4°C for 5 min, and the supernatant was removed for Western blot analysis. Gels were loaded according to the protein concentration assessed by the DC protein assay (Bio-Rad, CA), before Western blot aliquots of 2 μg/1 μL were prepared in 4× laemmli sample buffer and ddH_2_O. Before analysis, samples were left at room temperature overnight to denature, to maintain membrane integrity. Equal amounts of protein (18–30 μg) were loaded onto Criterion TGX Precast Midi protein gels (Bio-Rad) or homemade 12.5% protein gels, and separated by SDS-PAGE at a constant voltage of 100 V for 10 min and then 150 V for 1 h. Protein samples were then transferred at a constant voltage (100 V for 1 h) to a polyvinylidene difluoride (PVDF) or protran nitrocellulose membrane. The membranes were then incubated overnight at 4°C with a validated primary antibody; total OXPHOS human antibody cocktail [ab110411; 1:1,000 in 5% BSA:Tris-buffered saline-Tween 20 (TBST)], citrate synthase (CS; CST143095 1:1,000 in TBST), total acetyl-CoA carboxylase (ACC; CST3676 in TBST), p-ACC^S79^ (CST36615 in TBST), total 5′ AMP-activated protein kinase (AMPKα; CST2757, 1:1,000 in TBST), phospho-AMPK^αT172^ (CST2535, 1:1,000 in TBST), peroxisome proliferator-activated receptor gamma coactivator 1-α (PGC1α; MM3248419, 1:1,000 in 5% BSA:TBST), Calcium/calmodulin-dependent protein kinase type II (CAMKII; CST3362 1:500 in 5% BSA:TBST), PGC-1 and ERR-induced regulator in muscle protein 1 (PERM1; HPA031712, 1:500 in 5% BSA:TBST), mitochondrial transcription factor A (TFAM; SAB1401383 1:1,000 in 5% BSA: TBST), nitric oxide synthase (NOS; AB76198, 1:1,000 in 5% BSA:TBST), manganese superoxide dismutase (mnSOD; AB214675, 1:1,000 in 5% BSA:TBST), p-DRP1^S616^ (CST4494, 1:1,000 in TBST), total dynamin-related protein 1 (DRP1; CST5391, 1:1,000 in TBST), mitofusin 2 (MFN2; CST143095, 1:1,000 in TBST), mitochondrial fission factor (MFF; CST84580, 1:1,000 in TBST), p-MFF^S176^ (CST49281, 1:1,000 in TBST), total unc-51 like autophagy activating kinase (ULK1 CST4773, 1:1,000 in 5% BSA:TBST), p-ULK1S555 (CST5869 1:1,000 in 5% BSA:TBST), optic atrophy protein 1 (OPA1; BD Bioscience, 612607, 1:1,000 in TBST), mitochondrial fission 1 (FIS1; Atlas Antibodies, HPA017430, 1:1,000 in TBST), insulin receptor B (IR; CST23413, 1:1,000 in 3% BSA:TBST), total insulin receptor substrate (IRS; CST2390, 1:1,000 in 5% BSA:TBST), phosphoinositide 3-kinase (PI3K; CST4257, 1:1,000 in 5% BSA:TBST), total protein kinase B (Akt; CST9272, 1:1,000 in TBST), p-AktS473 (CST4060, 1:1,000 5% BSA in TBST), p-AktT308 (CST9275, 1:5,000 in TBST), glucose transporter type 4 (GLUT4; CST2213, 1:1,000 in TBST), total glycogen synthase kinase-3 (GSK3αβ; CST5676, 1:1,000 in TBST), p-GSK3αβ^S21/9^ (CST9331, 1:1,000 in TBST), total glycogen synthase (GS; CST3886, 1:1,000 in TBST), and p-glycogen synthase (p-GS^s641^; CST3886, 1:1,000 in TBST). Samples were then washed 3 × 5 min in TBST before undergoing a 1 h incubation with a previously validated horseradish peroxidase (HRP)-linked anti-rabbit (CST7074, 1:10,000 in TBST) or anti-mouse (CST7076, 1:10,000 in 5% BSA:TBST) IgG. Thereafter, immobilon western chemiluminescent HRP substrate (Millipore) was used quantify protein content, visualized using a BOX Chemi XT4 imager with GeneSys capture software (Syngene UK, Cambridge, UK). Quantification of bands was achieved using Chemi Genius Bioimaging Gel Doc System (Syngene, Cambridge, UK), and values were corrected to a loading control (ponceau). Where appropriate, the phosphorylation of proteins, as a proxy of their activation was expressed relative to the total amount of each protein. Data are presented as fold changes from the pre-SR condition.

### Citrate Synthase Activity Assay

Maximal CS enzyme activity was determined as previously described as a marker of mitochondrial content and adapted to 96-well microplate format for spectrophotometric analysis (38). Before measurement, sarcoplasmic homogenates were prepped at a concentration of 2 μg/μL ddH_2_O. CS reaction buffer [50 mM potassium phosphate (KPI) buffer; pH 7.4, 100 μM DTNB, and 115 μM acetyl-CoA in ddH_2_O], and spectrophotometer were warmed to 30°C for optimal enzymatic reactions. For baseline measurements, 10 μL (20 μg protein) of sample and 186 μL of warm reaction buffer were pipetted into a 96-well microplate, with a single participant measured at a time in triplicate. Baseline absorbance was read every 15 s for 3 min at 412 nm in a microplate reader (FLUOstar Omega, BMG Labtech, Aylesbury, UK). Immediately following this baseline measurement, 4 μL of oxaloacetate (100 μM final concentration) was added to each well to initiate the reaction before the plate was returned to the spectrophotometer and read again every 15 s for 3 min at 412 nm, to measure the rate of thionitrobenzoate anion (TNB) appearance. The protocol has previously been validated (38) and enzyme activity was calculated as: the Δ absorbance/min × 1,000/[(extinction coefficient × volume of sample used in mL) × (sample protein concentration in mg·mL^−1^)]. The average enzyme activity across three replicates was taken forward for analysis. The within-plate coefficient variation of the three technical replicates was 3.51 ± 2.51% and within the assay’s acceptable range, as previously reported (39).

### Statistics

Data are presented as means ± SE. Statistical assumptions were checked before analysis, and analysis was performed using SPSS statistics v. 25 (IBM Corp.). Measures of protein expression and enzymatic activity were assessed using a paired samples *t* test (pre-SR vs. post-SR). Missing data were not imputed and *n* numbers for each analysis are reported in figure legends. The level of significance was considered *P* ≤ 0.05.

## RESULTS

### Physical Activity and Dietary Intake

Changes in physical activity following step reduction have previously been published elsewhere (37). Briefly, daily step count was reduced by ∼91% during SR (13,054 ± 2,763 to 1,192 ± 330 steps/day; *P* < 0.001). The percentage of total time spent sedentary (73 ± 6 to 90 ± 3%; *P* < 0.001) increased, and percentage of total time spent standing (17 ± 6 to 8 ± 3%; *P* < 0.001) and ambulatory (10.0 ± 1.0 to 1.0 ± 0.5%; *P* < 0.001) decreased during SR. Finally, the number of daily transitions from a sitting to standing position were also significantly reduced during SR (46 ± 8 to 31 ± 10; *P* < 0.001). Dietary intake, which has been previously published (37), during habitual activity and SR is presented in Table 1.

**Table 1.** Dietary intake during habitual physical activity and step reduction

Variable	Habitual Physical Activity	Step Reduction
Energy intake, kcal·day^−1^	2,625 ± 732	2,380 ± 864
Protein, g·kg^−1^·day^−1^	2.1 ± 0.7	1.8 ± 0.6*
Protein intake, g·day^−1^	156 ± 51	133 ± 45*
Carbohydrate intake, g·day^−1^	297 ± 142	279 ± 165
Fat intake, g·day^−1^	83 ± 34	77 ± 33
Protein, En%	26 ± 13	24 ± 12
Carbohydrate, En%	46 ± 13	46 ± 12
Fat, En%	28 ± 9	29 ± 10

Values are means ± SD, *n* = 11. *Significant difference between habitual physical activity and step reduction conditions (*P* < 0.01).

### Mitochondrial Function

**Figure 1.**
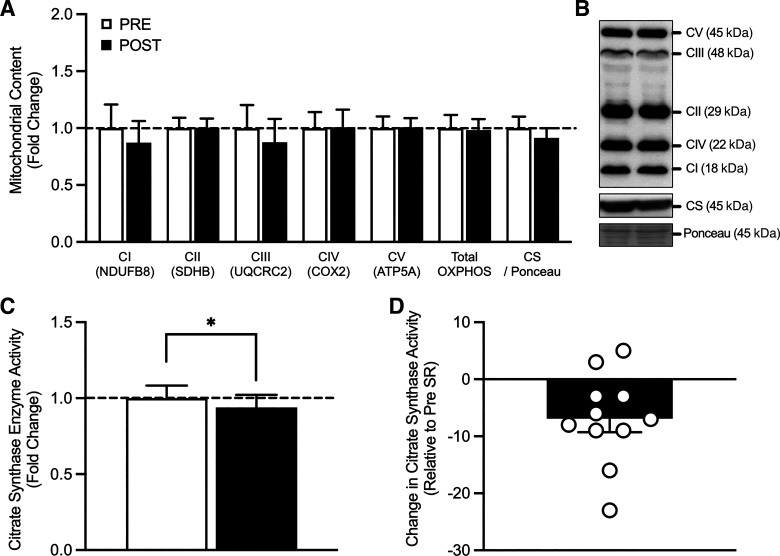
Protein content of proteins relating to mitochondrial function in response to 7 days step reduction in young males. OXPHOS CI, OXPHOS CII, OXPHOS CIII, OXPHOS CIV, OXPHOS CV, total OXPHOS protein content (*n* = 11), and citrate synthase protein content (*n* = 11; *A*), Western blot representative image (*B*), citrate synthase activity assay (*n* = 11; *C* and *D*). Data are presented as means ± SE and were analyzed using a repeated measures *t* test. *Post-SR was significantly different from pre-SR at the *P* < 0.05 level. CS, citrate synthase; *n*, number of subjects; OXPHOS, oxidative phosphorylation; SR, step reduction.

The expression of proteins of mitochondrial function following 7 days of SR can be viewed in Fig. 1, *A* and *B*. No significant alterations were noted following SR in OXPHOS CI protein content (fold change, pre vs. post: 0.87 ± 0.19, *P* = 0.492), OXPHOS CII protein content (1.00 ± 0.08, *P* = 0.938), OXPHOS CIII protein content (fold change, pre vs. post: 0.87 ± 0.21, *P* = 0.534), OXPHOS CIV protein content (fold change, pre vs. post: 1.01 ± 0.16, *P* = 0.935), OXPHOS CV protein content (fold change, pre vs. post: 1.01 ± 0.08, *P* = 0.873), TOTAL OXPHOS protein content (fold change, pre vs. post: 0.98 ± 0.09, *P* = 0.790), and CS protein content (fold change, pre vs. post: 0.91 ± 0.08, *P* = 0.267). In contrast, maximal CS activity, a marker of mitochondrial content, significantly reduced following a 7-days period of SR (Fig. 1, *C* and *D*; fold change, pre vs. post: 0.94 ± 0.08, *P* = 0.012).

### Oxidative Metabolism

**Figure 2.**
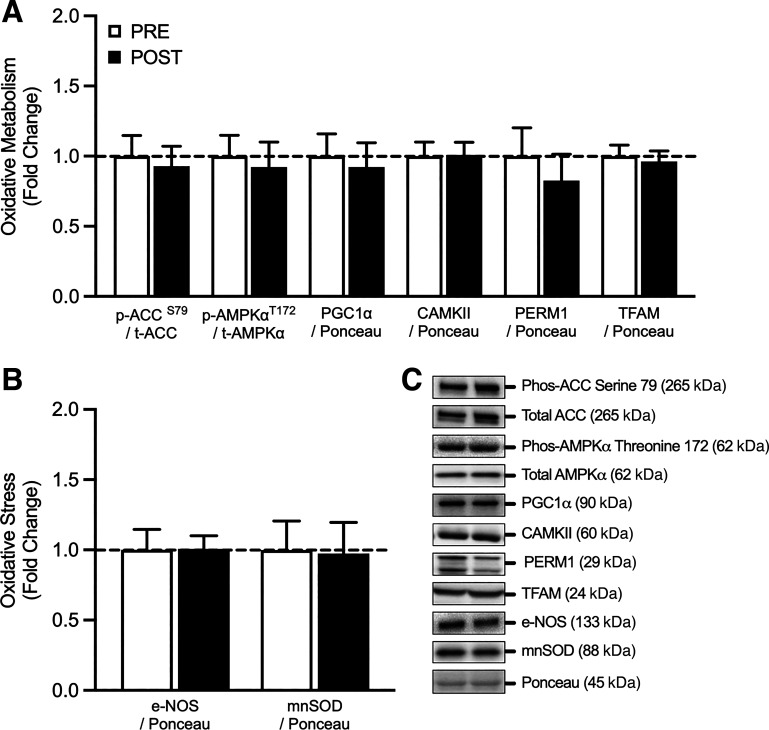
Total and phosphorylated protein content of proteins relating to oxidative metabolism (*n* = 11; *A*) and oxidative stress (*n* = 11; *B*) in response to 7 days step reduction in young males. *C*: illustrates a representative Western blot image of total and phosphorylated protein content of proteins relating to oxidative metabolism and oxidative stress markers. Data are presented as means ± SE and were analyzed using a repeated measures *t* test. ACC, acetyl-CoA carboxylase; AMPKα, 5′ AMP-activated protein kinase; mnSOD, manganese superoxide dismutase; *n*, number of subjects; NOS, nitric oxide synthase; PGC1α, peroxisome proliferator-activated receptor gamma coactivator 1-α; SR, step reduction.

Expression of key markers of oxidative metabolism and oxidative stress can be seen in Fig. 2, *A* and *B*, respectively. There were no significant differences in PCG1α protein content (fold change, pre vs. post: 0.92 ± 0.17, *P* = 0.514), PERM1 protein content (fold change, pre vs post: 0.83 ± 0.19, *P* = 0.074), CAMKII protein content (fold change, pre vs. post: 1.00 ± 0.08, *P* = 0.845), or TFAM protein content (fold change, pre vs. post: 0.96 ± 0.07, *P* = 0.265) following 7-days SR. Furthermore, the activation (or phosphorylation) of AMPKα^T172^ (fold change, pre vs. post: 0.92 ± 0.18, *P* = 0.597) and ACC^S79^ (fold change, pre vs. post: 0.93 ± 0.14, *P* = 0.523) was not significantly different following the SR intervention. Finally, there were no significant differences in the protein content of mnSOD (fold change, pre vs. post: 0.97 ± 0.22, *P* = 0.840) or NOS (fold change, pre vs. post: 1.01 ± 0.09, *P* = 0.942) following 7 days of SR.

### Mitochondrial Dynamics

**Figure 3.**
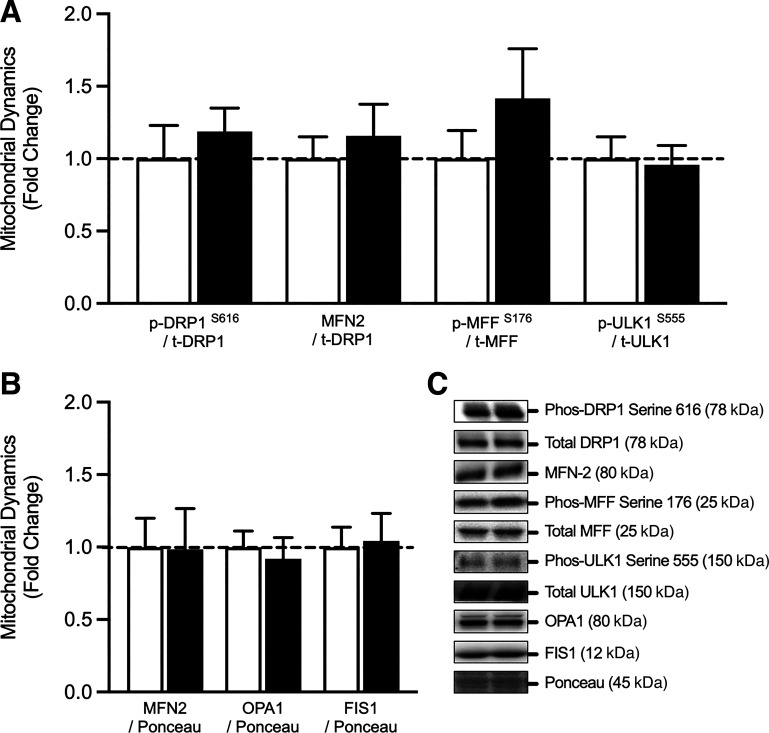
Total and phosphorylated protein content of proteins relating to mitochondrial dynamics. *A* and *B*: (*n* = 11) in response to 7 days step reduction in young males. *C*: illustrates a representative Western blot image of total and phosphorylated protein content relating to mitochondrial dynamics. Data are presented as means ± SE and were analyzed using a repeated measures *t* test. DRP1, dynamin-related protein 1; FIS1, mitochondrial fission 1; MFF, mitochondrial fission factor; MFN2, mitofusin 2; *n*, number of subjects; OPA1, dynamin-like 120 kDa protein; ULK1, unc-51 like autophagy activating kinase.

In response to 7 days of SR, no significant differences were noted in the expression or activation of proteins involved in mitochondrial fission or fusion (Fig. 3, *A* and *B*). Specifically, the expression of FIS1 protein content (fold change: pre vs. post: 1.04 ± 0.19, *P* = 0.516), MFF protein content (fold change: pre vs. post: 1.42 ± 0.34, *P* = 0.152), MFN2 protein content (fold change, pre vs. post: 0.98 ± 0.28, *P* = 0.923), and OPA1 protein content (fold change, pre vs. post: 0.92 ± 0.15, *P* = 0.329) remained unchanged following SR. Likewise, the activation (or phosphorylation) of DRP^S616^ (fold change, pre vs. post: 1.19 ± 0.16, *P* = 0.223) and ULK1^S555^ (fold change, pre vs. post: 0.96 ± 0.13, *P* = 0.829) were not significantly different following 7 days of SR. Finally, the ratio of MFN to total-DRP1 protein content (fold change, pre vs. post:1.16 ± 0.22, *P* = 0.141) remained unchanged.

### Glucose Metabolism

**Figure 4.**
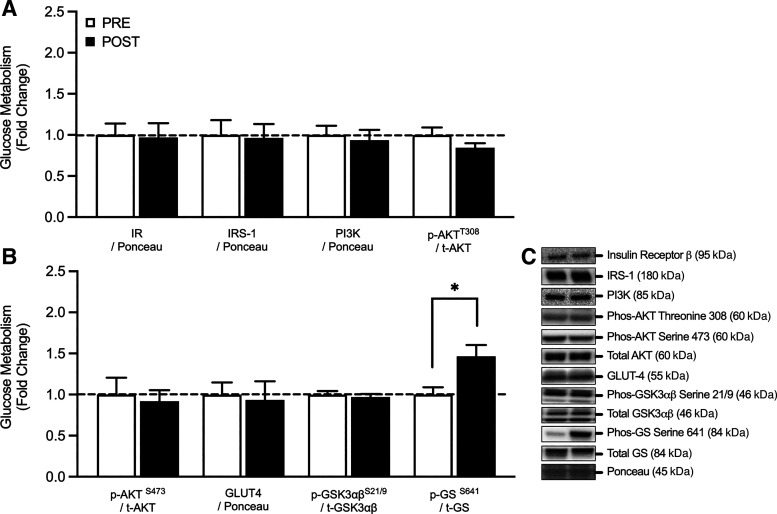
Total and phosphorylated protein content of proteins relating to skeletal muscle glucose metabolism (*n* = 11) in response to 7 days step reduction in young males (*A* and *B*). *C*: illustrates a representative Western blot image of total and phosphorylated protein content relating to skeletal muscle glucose metabolism. Data are presented as means ± SE and were analyzed using a repeated measures *t* test. *Post-SR was significantly different from pre-SR at the *P* < 0.05 level. AKT, protein kinase B; GLUT4, glucose transporter 4; GS, glycogen synthase; GSK3αβ, glycogen synthase kinase-3αβ; IR, insulin receptor B; IRS, insulin receptor substrate; *n*, number of subjects; PI3K, phosphoinositide 3-kinase.

Alterations of key markers of skeletal muscle glucose metabolism are presented in Fig. 4. In response to 7 days of SR, there was a significant increase in the activation (or phosphorylation) of GS^S641^ (fold change, pre vs. post: 1.47 ± 0.14, *P* = 0.012). There were no further significant differences noted in the phosphorylation of GSK3αβ^S21/9^ (fold change, pre vs. post: 0.97 ± 0.04, *P* = 0.486), AKT^S473^ (fold change, pre vs. post: 0.92 ± 0.13, *P* = 0.520), or AKT^T308^ (fold change, pre vs. post: 0.84 ± 0.06, *P* = 0.161) following the 7 days intervention. Similarly, the protein content of IR (fold change, pre vs. post: 0.97 ± 0.17, *P* = 0.882), IRS (fold change, pre vs. post: 0.96 ± 0.17, *P* = 0.738), PI3K (fold change, pre vs. post: 0.94 ± 0.13, *P* = 0.278), and GLUT4 (fold change, pre vs. post: 0.93 ± 0.23, *P* = 0.558) remained unchanged following 7 days of SR.

## DISCUSSION

We report that in response to 7 days of SR in young, healthy males there was a significant decline in maximal citrate synthase (CS) activity, a marker of mitochondrial content. Despite this alteration in maximal CS activity, no significant declines in the total protein or phosphorylation content of markers of mitochondrial function (e.g., OXPHOS complex I-V), oxidative metabolism (e.g., PGC1α, AMPKα), or mitochondrial dynamics (e.g., FIS1, DRP1, and MFN2) were noted in response to 7-day SR. Due to the purported link between alterations in mitochondrial metabolism and fuel utilization during musculoskeletal disuse, we also examined the expression and phosphorylation of proteins involved in skeletal muscle insulin sensitivity. We found a significant increase in p-GS^S641^/t-GS in response to 7-day SR. However, no additional changes in total or phosphorylated protein content of markers of insulin sensitivity (e.g., IR, AKT, and GLUT4) were noted.

Previous work in severe models of musculoskeletal disuse (i.e., bed rest/limb immobilization) in young healthy individuals has demonstrated a significant decline in CS activity (7, 25, 40, 41), which is accompanied by declines in the expression of CS and the OXPHOS complex proteins (7, 11, 25, 42). Despite previous evidence of compromised mitochondrial function following a short-term period (7–14 days) of severe musculoskeletal disuse, whether the reduced loading and energetic demand of 7 days of SR would alter mitochondrial functioning was unknown. Here, we report for the first time that a significant decline in maximal CS activity occurred following 7 days of SR, which was not accompanied by alterations in the protein content of CS or OXPHOS CI-V. The reduction in maximal CS activity is perhaps unsurprising, as CS is an important regulator of the citric acid cycle and is inhibited under conditions of a high-energy supply (43). Though, this is still an important finding. Indeed, maximal CS activity is considered a robust marker of mitochondrial content (albeit not function, per se) and thus it is remarkable that simply reducing steps over a 7-day period, reduces maximal CS activity and, thus potentially mitochondrial content. During periods of SR, there is a reduction in contractile activity and likely a subsequent reduction in the requirement for ATP synthesis. Indeed, it is likely that energy intake may exceed skeletal muscle energetic demand throughout the period of reduced ambulation, resulting in the “underutilization” of ATP. Since high ATP concentration allosterically inhibits CS (44), it is plausible that the reduction in contractile activity (and thus ATP usage) would reduce the saturation of this enzyme with acetyl-CoA and subsequently dampen its activity and total mitochondrial content. Interestingly, and in contrast to our hypotheses, any shift in energy utilization and enzymatic activity/mitochondrial content was not severe enough to promote alterations in the expression of CS or OXHPOS CI-V over 7 days of SR. This finding is also in contrast to previous literature in models of bed rest/limb immobilization (11, 25, 42) and suggests that the complete removal of contractile stimuli and potentially more drastic alterations in physical activity may be required to alter protein content in a young population over a 7-day period. Due to the retrospective sample analysis of a larger experimental trial, and thus lack of fresh tissue for analysis, we were unable to determine the OXPHOS respiratory capacity, which remains a limitation of these data and provides rationale for further research to measure intramuscular ATP and general energy utilization across disuse models to fully resolve this question. Further, it is worthy of note that the current study does not include a “true” control condition, such that we did not include a parallel group without the physical inactivity intervention, and thus cannot discount any time effects independent of the inactivity intervention (effect of time per se, repeated biopsies etc.). Nevertheless, our data does provide novel insights into the compensatory declines in CS enzymatic activity (and potentially mitochondrial content) following as little as 7-day SR in young adults, without associated alterations in the expression of key markers of mitochondrial function.

The complete removal of contractile stimuli (e.g., bed rest) has been shown to trigger a cascade of alterations in the expression of key signaling intermediates of oxidative metabolism (11, 16, 45, 46), ultimately leading to a reduced rate of mitochondrial synthesis (11, 24). Here, we hypothesized that SR would adversely affect the expression and phosphorylation of key signaling proteins involved in oxidative metabolism (e.g., AMPKα, PGC1α, ACC, and TFAM) and Ca^2+^ handling (e.g., CAMKII and PERM1). In contrast to previous studies (11, 16, 45, 46), we reported no alterations in the total expression or phosphorylation of proteins involved in oxidative metabolism. Similarly, there was no significant difference in the CAMKII or PERM1 protein expression, which contrasted with previous reports in catabolic conditions (47, 48). Taken together, these data suggest that any alterations in Ca^2+^ and AMP:ATP that may occur during 7 days of SR may not be severe enough to potentiate alterations in mitochondrial biogenesis, whereas complete removal of contractile activity may instigate such adverse metabolic responses, at least in young healthy individuals.

Alterations in oxidative metabolism and Ca^2+^ handling as a result of musculoskeletal disuse (49–51) are linked to increases in ROS production, through the stimulation of the citric acid cycle and subsequent activation of ROS generating enzymes (52, 53). Our data may suggest that 7 days of SR does not provide a robust enough “unloading stimulus” to significantly alter these parameters, and in combination with the reduction in maximal CS activity, may explain why we did not detect any significant alterations in MnSOD or NOS expression following the 7 days of SR. Furthermore, the generation of ROS following musculoskeletal disuse has been putatively linked to myofibrillar protein imbalance and the subsequent onset of muscle atrophy (27). However, following the 7-day period of SR in the current intervention, declines in muscle protein synthesis in combination with increased gene expression of catabolic signaling targets [see Ref. Shad et al. (37) for these previously published data], occurred independently of alterations in markers of mitochondrial function and ROS production. Taken together, these data lead us to speculate that with 7 days of SR in young individuals, alterations in myofibrillar protein turnover [as previously published (37)] occur independently from alterations in the abundance of mitochondrial proteins.

Mitochondrial morphology is dependent on rates of mitochondrial fusion and fission (27). Preclinical models suggest a shift in mitochondrial dynamics toward fission (28), following a period of immobilization, resulting in an increase of fragmented mitochondria (29–31). However, this is yet to be consistently demonstrated in human disuse studies. Here, we report no significant alterations in the total or phosphorylated protein content of markers of mitochondrial fission or fusion following 7 days of SR in young healthy males. Alterations in mitochondrial dynamics occur in response to cellular stress (54), thus the lack of significant changes noted in the expression of markers of mitochondrial dynamics herein, further suggests that 7 days of SR does not significantly impact on cellular energy homeostasis. This finding is in line with previous work (45), in which no differences in the content of mitochondrial fission or fusion proteins was reported following 10 days of best rest. These data shed further light on the discrepant alterations in mitochondrial dynamics between animal and human models of disuse atrophy.

The lack of alterations in total protein and/or phosphorylated protein content of proteins involved in mitochondrial metabolism and energy homeostasis may explain why we did not observe any alterations in the total expression or phosphorylation of signaling intermediates of skeletal muscle glucose uptake (i.e., IR, IRS, Akt, and GLUT4) following 7 days of SR. However, we did note a significant increase in P-GS^S641^/t-GS following the intervention, which is a marker of reduced GS activation. In response to muscular contraction, there is a reduction in GS phosphorylation, promoting an increase in GS activity (55), so it is perhaps unsurprising that a reduction in contractile activity promoted an increase in GS phosphorylation. This finding may also explain, in part, the mechanisms underpinning the significant decline in whole body insulin sensitivity noted in this cohort of participants following the 7 days SR intervention [see (37) for additional data]. Importantly, a decline in glycogen content is key to maintaining skeletal muscle insulin sensitivity. Since a high-glycogen content reduces GS activity, it is possible that the increase in GS phosphorylation (which can reduce GS activity) noted here, may represent a protective mechanism to maintain insulin sensitivity toward homeostatic levels during a period of reduced ambulation (56). The regulation of GS activity is dependent on various kinases including GSK3, CAMKII, and AMPK (57). However, we did not note any significant differences in the protein content or phosphorylation of these signaling targets, suggesting that, at least in the current cohort, alterations in the phosphorylation of GS occurred independently to alterations in total protein content and/or phosphorylation of proteins involved in oxidative metabolism and ATP synthesis. Though, it is pertinent to note that a limitation of the current study is that no muscle biopsies were taken under insulin-stimulated conditions and thus the conclusions that can be drawn are limited.

In conclusion, 7 days of SR in young males caused significant declines in maximal CS activity (a marker of mitochondrial content), independent to alterations in the total protein content or phosphorylation of key markers involved in mitochondrial function, oxidative metabolism, and mitochondrial dynamics. Furthermore, following the 7-days SR intervention, there was a significant increase in the phosphorylation of GS, which occurred independently to additional alterations in the expression of markers involved in glucose uptake. These data provide a further resolution to suggest declines in myofibrillar protein synthesis, demonstrated in our previous publication, in response to 7 days SR occur independently to alterations to the expression of key markers involved in oxidative protein metabolism and glucose uptake in young healthy males.

## GRANTS

This work was supported by a studentship (to S.J.E) from the BBSRC Midlands Integrative Biosciences Training Partnership and a Exercise as Medicine studentship (to B.J.S) from the College of Life and Environmental Sciences, University of Birmingham.

## DISCLOSURES

No conflicts of interest, financial or otherwise, are declared by the authors.

## AUTHOR CONTRIBUTIONS

S.J.E., B.J.S., G.A.W., and L.B. conceived and designed research; B.J.S. and G.A.W. performed experiments; S.J.E., R.N.M., P.T.M., and G.A.W. analyzed data; S.J.E., P.T.M., and L.B. interpreted results of experiments; S.J.E. prepared figures; S.J.E. and L.B. drafted manuscript; S.J.E., B.J.S., R.N.M., P.T.M., G.A.W., and L.B. edited and revised manuscript; S.J.E., B.J.S., R.N.M., P.T.M., G.A.W., and L.B. approved final version of manuscript.
